# Editorial: Autoimmunity and cardiovascular diseases

**DOI:** 10.3389/fcvm.2023.1249525

**Published:** 2023-07-18

**Authors:** Shahad Saif Khandker, Przemysław J. Kotyla, Kamarul Imran Musa, Md Asiful Islam

**Affiliations:** ^1^ Department of Microbiology, Gonoshasthaya Samaj Vittik Medical College, Savar, Dhaka Division, Bangladesh; ^2^ Department of Internal Medicine, Rheumatology and Clinical Immunology, Faculty in Katowice, Medical University of Silesia, Katowice, Poland; ^3^ Department of Community Medicine, School of Medical Sciences, Universiti Sains Malaysia, Kubang Kerian, Malaysia; ^4^ WHO Collaborating Centre for Global Women’s Health, Institute of Metabolism and Systems Research, University of Birmingham, Birmingham, United Kingdom

**Keywords:** autoimmune, cardiovascular, autoantibody, cytokine, immune cell

**Editorial on the Research Topic** Autoimmunity and cardiovascular diseases

Cardiovascular disease (CVD) is one of the most severe complications of several diseases such as type 2 diabetes mellitus, hypertension, hyperlipidemia, high cholesterol, and many others (1). Besides, autoimmunity and the enhancement of autoantibodies strongly correlate with CVD development (2, 3). Epidemiological studies have shown that the autoimmune diseases that are risk factors for CVD can be fatal or responsible for a significant reduction in the lifespan of affected individuals (2).

Rheumatoid Arthritis, one of the most common inflammatory arthropathy in humans, is linked with an increased risk of cardiovascular events, such as the association of anti-cyclic citrullinated peptide with ischemic cardiac arrest (4). Additionally, systemic lupus erythematosus (SLE) is also associated with a significantly increased risk of cardiovascular morbidity and mortality. The unquestioned pioneer in the field of CVD and autoimmune disorders, Urowitz et al. coined the term “bimodal mortality pattern of mortality in SLE” (5). This theory relates the significant reduction in life expectancies of SLE patients with associated cardiovascular events and their fatal complications.

Given the association of inflammation with autoimmunity, the unusual production of cytokines may also be a substantial risk factor in the development of CVD (6). In animal models, pro-inflammatory Th-1 cytokines, such as IFN-γ, were previously perceived to be atherogenic, though is supported with limited data (7). Further research is required to understand the correlation between autoimmunity and CVD.

This special issue aimed to create a forum to highlight novel insights into autoimmunity and CVD and provide readers with a broad overview on the current and emerging research in this field. As a result, this issue has been enriched with several outstanding original articles with diverse research concepts; nevertheless, keeping the ideas aligned with the topic.

Initially, the article we received was entitled “single-cell transcriptomes and T cell receptors of vaccine-expanded apolipoprotein B-specific T cells” by Nettersheim FS et al. where they administrated ApoB-Peptide P6 (ApoB978−993 TGAYSNASSTESASY) vaccine in C57BL/6J mice model. Consequently, single-cell RNA sequencing of tetramer-sorted P6+ T cells was performed which were further clonally expanded (one major, two minor clones) and formed a transcriptional cluster distinct from clusters mainly containing non-expanded P6^+^ and P6^−^ cells. The study investigated the oligoclonal expansion of CD4 T cells due to the ApoB-P6 vaccination. The major portion of clonally expanded cells expressed a proper T_reg_ signature and demonstrated an upregulation of genes that are involved in mediating the suppressive function. The efficacious renovation of TCR*α* and TCRβ in most of the cells, combined with known peptide epitope and sorted with P6:I-A^b^ tetramer, displays complete structural information on an ApoB-specific TCR with peptide-loaded MHC-II. This study would enlighten further research on vaccination strategies especially with ApoB to modify proatherogenic autoimmunity.

Another article we received was entitled “the gut microbiota in experimental abdominal aortic aneurysm” authored by Jie Xiao et al. Here, the author tried to explore the plausible association between gut microbiota and the formation of Abdominal aortic aneurysm (AAA). Xiao et al. used a mouse model and administrated classical angiotensin-II to induce AAA in mice. Mice were divided into 2 groups (i.e., control and AAA) and the treatment continued for 28 consecutive days. Aortic and fecal samples were collected from where fecal samples were analyzed by 16s rRNA sequencing. As a result, they found that *Akkermansia muciniphila, Allobaculum,* and *Barnesiella intestinihominis* species were increased in the control group whereas *Oscillospira, Coprococcus, Faecalibacterium prausnitzii, Alistipes massiliensis,* and *Ruminococcus gnavus* were increased in the AAA group. Further, the network, as well as PICRUSt2 analysis and Zipi score assessment, indicated that the metabolic pathways such as PWY-6629 (a super pathway of L-tryptophan biosynthesis), PWY-7446 (sulfoglycolysis), and PWY-6165 chorismate biosynthesis II (archaea) may be involved in the formation of AAA which was plausibly induced by the *E. coli* and/or *Shigella*.

The clinical study of Wang et al. entitled “relationship between rheumatoid arthritis and cardiovascular comorbidity, causation or co-occurrence: a mendelian randomization study” (Wang et al.) assessed the possible association between different forms of heart disease (HD) and rheumatoid arthritis (RA). The Authors considered myocardial infarction (MI), arrhythmia, atrial fibrillation (AF), and ischemic heart disease (IHD) datasets obtained from the genome-wide association study dataset, and the mendelian randomized estimates were calculated with Inverse-variance weighted method. The result identified that the genetic susceptibility of RA was remarkably correlated with MI and IHD as compared to the correlation of RA with arrhythmia and AF. The findings indicate that the minimization of RA development might inhibit the risk of HD.

The three articles exhibited some exclusive and impactful ideas which can be food for thought for the researchers to move forward and uncover the veils of unknown molecular pathways and mechanisms between autoimmune and cardiovascular diseases for better understanding.

## References

[1] Assmann G Schulte H. Diabetes mellitus and hypertension in the elderly: concomitant hyperlipidemia and coronary heart disease risk. Am J Cardiol. (1989) 63(16):33–7. 10.1016/0002-9149(89)90113-6 2650522

[2] Conrad N Verbeke G Molenberghs G Goetschalckx L Callender T Cambridge G Autoimmune diseases and cardiovascular risk: a population-based study on 19 autoimmune diseases and 12 cardiovascular diseases in 22 million individuals in the UK. Lancet. (2022) 400(10354):733–43. 10.1016/S0140-6736(22)01349-6 36041475

[3] Kotyla PJ Islam MA Engelmann M. Clinical aspects of Janus kinase (JAK) inhibitors in the cardiovascular system in patients with rheumatoid arthritis. Int J Mol Sci. (2020) 21(19):7390. 10.3390/ijms21197390 33036382 PMC7583966

[4] Abdel Wahab MAK Laban AE Hasan AA Darweesh AF. The clinical value of anti–cyclic citrullinated peptide (anti-CCP) antibodies and insulin resistance (IR) in detection of early and subclinical atherosclerosis in rheumatoid arthritis. The Egyptian Heart Journal. (2016) 68(2):109–16. 10.1016/j.ehj.2015.01.001

[5] Urowitz MB Bookman AAM Koehler BE Gordon DA Smythe HA Ogryzlo MA. The bimodal mortality pattern of systemic lupus erythematosus. Am J Med. (1976) 60(2):221–5. 10.1016/0002-9343(76)90431-9 1251849

[6] Kofler S Nickel T Weis M. Role of cytokines in cardiovascular diseases: a focus on endothelial responses to inflammation. Clin Sci. (2005) 108(3):205–13. 10.1042/CS20040174 15540988

[7] Elyasi A Voloshyna I Ahmed S Kasselman LJ Behbodikhah J De Leon J The role of interferon-γ in cardiovascular disease: an update. Inflammation Res. (2020) 69:975–88. 10.1007/s00011-020-01382-6 32699989

